# Decision optimization of emergency material support based on blockchain under major public health emergencies

**DOI:** 10.1038/s41598-022-12819-9

**Published:** 2022-06-01

**Authors:** Hanyi Wang, Chuanzhang Fan, KunBao

**Affiliations:** School of Economics and Management, Kunming University, Kunming, 650214 China

**Keywords:** Physics, Health care

## Abstract

The work intends to relieve the pressure on the urban medical system and reduce the cross-infection of personnel in major public health emergencies. On the premise of an in-depth analysis of the utility risk entropy algorithm model and prospect theory, the decision-making of major health emergencies is proposed. Firstly, the utility risk entropy algorithm model is optimized, and the main decision-making members are subjected to utility perception according to the perceived utility values of different levels of risk, and the weights of decision-making members are calculated and revised according to the results of utility clustering. Secondly, the prospect theory is optimized. Taking the zero as the reference point to calculate the prospect value, and taking the maximization of the comprehensive prospect value as the objective to optimize the model, the comprehensive prospect value of each scheme is calculated and sorted. Finally, the proposed scheme is tested, and the test results show that in the optimal decision-making time of the scheme, the optimal decision-making time is 0 every day. When the epidemic situation is in the first cycle, the decision-making loss of the optimal scheme is 2.69, and the reduction ratio of the optimal scheme decision-making loss is 63.96%. When the epidemic situation is in the second cycle, the decision-making loss of the optimal scheme is 0.65, and the reduction ratio of the optimal scheme decision-making loss is 94.44%. When the epidemic situation is in the third cycle, the decision-making loss of the optimal scheme is 0.22, and the reduction ratio of the decision-making loss of the optimal scheme is 89.39%. The proposed scheme can improve the processing efficiency of major health emergencies and reduce the risk of accidents.

## Introduction

With the continuous development of science and technology and the continuous improvement of the economic level, the complexity and uncertainty of our society are increasing. This ever-increasing complexity and uncertainty provide the necessary conditions for the occurrence of unexpected events. The outbreak of COVID-19 in 2019 has caused great harm to people's health and social-economic development all over the world. Medical protective articles, medicines, and medical equipment are the basis of epidemic prevention and control. However, medical protective substances in various countries suffering epidemics are often insufficient, in this epidemic prevention and control work. This reflects that there are some shortcomings and defects in somatic cells of emergency supplies in various countries suffering epidemics^1,2^. In the process of multi-scenario risk decision-making, the decision-maker has a certain prediction on the attribute value, which is completely rational expected utility, but the decision-maker, in reality, is finitely rational^3,4^.

Researchers also put forward optimization models for these shortcomings. Ling et al.^5^ pointed out that in large-scale epidemics such as novel coronavirus (COVID-19), medical masks, respirators, sickbeds, and other medical supplies were in great demand. Resources from civilian medical services were often insufficient to fully meet all these needs. Military medical service resources usually reserved for military use could effectively supplement these demands. The problem is proposed for military-civilian integrated medical material scheduling for epidemic prevention and control. The solvent of this problem is to minimize the total scheduling cost while maximizing the overall satisfaction rate of medical materials. While keeping a minimum proportion of medical supplies for military use, to solve this problem effectively, an algorithm is proposed for multi-objective water wave optimization. The calculation results of a group of problem examples based on real COVID-19 data prove the effectiveness of this method. Li et al.^6^ pointed out that unexpected events such as natural disasters and other public disasters seriously threatened the safety of people's lives and property all over the world. For this reason, an in-depth study is made on the allocation of emergency materials. Initially, the study is made on the location-route problem of alternative logistics centers and material demand points, and the establishment is completed on a multi-objective integer programming model according to the actual situation. The model includes two objectives: (1) the minimum total transportation time; (2) the maximum value of total satisfaction of emergency materials. Then an algorithm for solving the above model is introduced. Finally, verification is made on the applicability and effectiveness of the above methods and models. Turke and Srensen^7^ pointed out that more and more studies had been done to improve the disaster preparedness capacity by pre-positioning relief materials in strategic locations, but there was a lack of a set of benchmarks for example. It hindered comprehensive hypothesis testing, sensitivity analysis, model verification, or evaluation of solution procedures. The research purpose is to solve this problem by setting up a different reservation example of the public library. A tool is developed to generate any number of different random instances of any size by algorithm, The scheme proposed can be used to study the pre-positioning and related, or to obtain the management impact that can directly benefit practitioners. These examples of social impact can be used to derive practical guidelines, which can also be adopted by humanitarian workers in the field to better scheme the pre-deployment strategy and minimize human suffering. Case studies and random case generators are made public to promote further research on pre-deployment of relief materials and humanitarian logistics. Liu et al.^8^ proposed a multi-objective optimization model for emergency material allocation with continuous time-varying supply and demand constraints, to minimize the losses and economic costs of emergency rescue operations. Sequential unconstrained minimization technology is adopted to deal with constrained optimization problems, fast non-dominated sorting genetic algorithm is adopted for multi-objective optimization, and elite strategy is adopted to obtain Pareto solution set with fairness and balance of loss and cost to find a suitable non-inferior solution and its corresponding material distribution scheme. The scheme put forward provides decision support for the continuous allocation of emergency relief materials. Yuan et al.^9^ pointed out that in the face of various emergencies, emergency logistics vehicles were needed to meet the needs of the affected areas in a short enough time. However, due to the suddenness of the incident and the shortage of relief materials, under the condition of insufficient materials, how to optimize the running route of emergency vehicles is a problem that needs further consideration. The corresponding mathematical model is established together with a genetic algorithm to solve the relevant examples, which provides a new solution method for the emergency logistics vehicle routing problem when the relief materials are insufficient. According to the analysis results of an example, the effectiveness of the optimization method is further demonstrated, which provides theoretical support for relevant decision-makers.

However, there are still some deficiencies in the research angle and research results of the researchers. Therefore, the risks of different stages of the development and evolution of major public health emergencies are analyzed. According to the perceived utility value of different levels of risk, the main decision-making members are perceived as utility, and a utility matrix is established. The weight of decision-making members is calculated and revised according to the results of utility clustering, and the decision-making scheme is determined according to the clustering results. The risk decision-making model is constructed by combining the utility risk entropy algorithm and prospect theory. The model is used to conduct case analysis, calculate the conflict risk entropy of different types of health safety accidents. Combined with utility perception, the emergency plan is prioritized, thereby improving the processing efficiency of major health emergencies and reducing the risk of accidents.

The innovation can be divided into two points. The first point is to analyze and summarize the structure characteristics of decision information in the process of multi-scenario risk decision-making. Aiming at the complex situation in which decision makers' expectations and attribute values are mixed with certain numbers, interval numbers and language phrases, a new multi-scenario mixed risk decision-making method based on utility risk entropy is proposed. The second point is to effectively combine risk probability and utility value function in the process of analyzing and summarizing multi-scenario risk decision-making, and on this basis, the measurement criterion of utility risk entropy is given.

The development status of decision analysis for major health emergencies is described in the Introduction; the construction of the utility risk entropy algorithm for clustering optimization is described in the “Research on utility risk entropy algorithm based on clustering optimization”, and *the multi-attribute decision-making problem of the number of three-parameter intervals is solved; the basic situation of the epidemic case analysis is described in the “Example analysis”, and the COVID-19 case is analyzed. The research content is summarized in the “Conclusions”.

## Research on utility risk entropy algorithm based on clustering optimization

### Analysis of utility entropy algorithm

The risk of a decision-maker taking an action depends on the expected utility of the action taken by the decision-maker. The definition of expected utility-entropy risk is as follows: given a decision-making problem of risk type $G = (\Theta ,A,u)$, the meaning of $\Theta$ is the state space of possible states, $A$ is the action space, $u$ is the utility function^10,11^, and the action plan is $a \in A$. When the relationship exists and is not 0, as shown in Eq. (1):

1 $$ \mathop {\max \{ \left| {E[uX(a,c)]} \right|\} }\limits_{a \in A} $$

The definition of expected utility-entropy risk using the action plan $a$ is shown in Eq. (2):

2 $$ R(a) = H_{a} (\theta ) $$

When $\mathop {max\{\left| {E[u(X(a,\theta ))]} \right|\} }\limits_{a \in A}$ equals 0, and meets that the action scheme $a \in A$, Eq. (3) can express the relationship.

3 $$ R(a) = H_{a} (\theta ) $$

In Eq. (3), $R(a)$ means the risk of action $a$, and $H_{a} (\theta )$ stands for the entropy of the corresponding state $\theta$ of action $a$.

**Figure 1:**
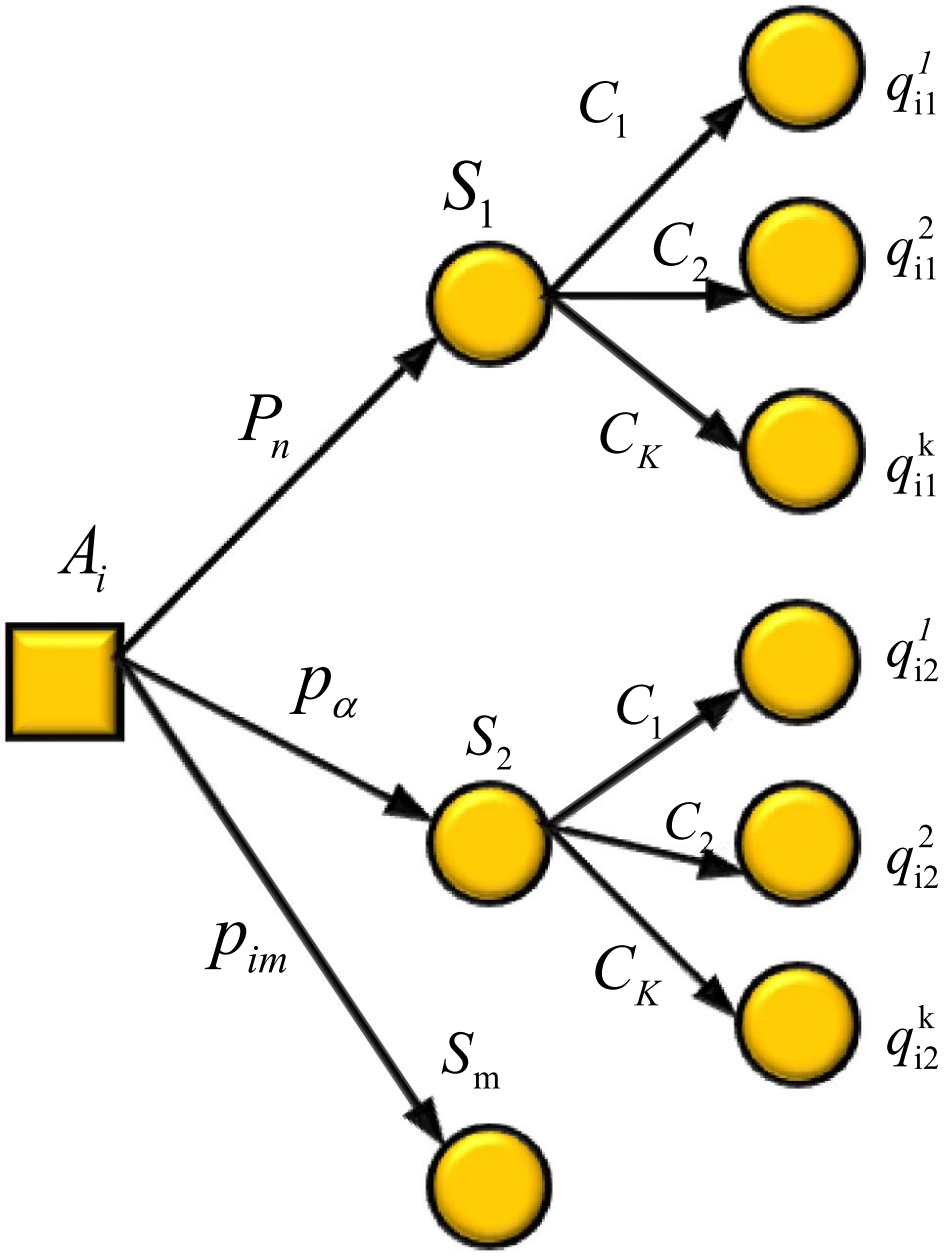
Multi-scenario mixed risk decision-making problem framework.

**Figure 2:**
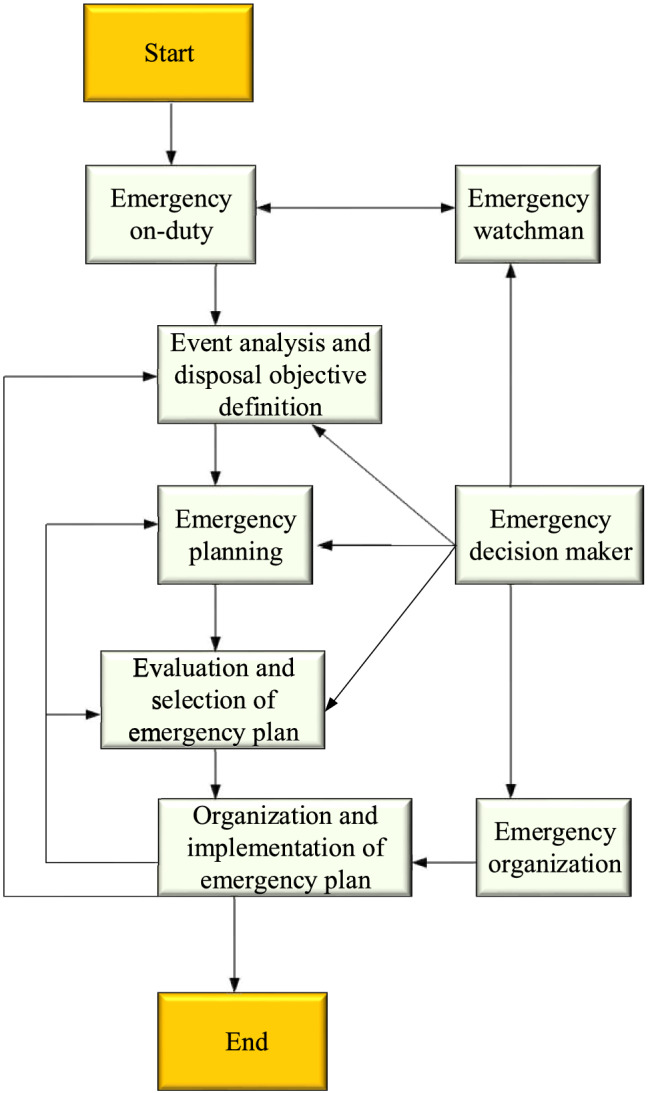
Sequence diagram of public emergency decision-making process.

According to the related definitions, it is not difficult to find that the expected utility-entropy decision model is defined as follows: $a_{1}$, $a_{2}$ refer to two action schemes in A; The expected utility-entropy risk measure is expressed as: $R(a_{1} )$, $R(a_{2} )$. In the expected utility-entropy risk measurement and risk decision model, the expected utility-entropy balance coefficient is very important, and the difference of expected utility-entropy balance coefficient will lead to the difference of expected utility-entropy risk $R(a)$, and then lead to different decision results, which cause the loses of the effectiveness of the model^12,13^. Figure 1 shows the framework of multi-scenario mixed risk decision-making. Figure 2 displays the sequence of emergency decision-making process for public emergencies.

In Fig. 1, $A_{i}$ is the set of alternatives, $S_{m}$ is the set of possible scenarios in the event's final state, $C_{k}$ is the attribute set under different scenarios, $P_{n}$ is the probability matrix of the result scenarios, and $q_{{{\text{i1}}}}^{1}$ is the result.

### Two-parameter interval number and three-parameter interval number

For arbitrary $$a{}^{L}$$, $$a^{R}$$$$\in$$$$R$$, if $$a^{L}$$$$\le$$$$a^{R}$$, $$a = [a^{L} ,a^{R} ]$$ can be called the two-parameter interval number. The midpoint value of the two-parameter interval number reflects the degree of data concentration, and the radius measures the degree of uncertain information. The two-parameter interval number can be regarded as a set containing exact numbers, which contains three relationships^14,15^. If $$a = \left[ {a^{L} ,a^{R} } \right]$$, $$b = \left[ {b^{L} ,b^{R} } \right]$$, and $$0 \le b^{L} \le b^{R}$$, $$0 \le b^{L} \le b^{R}$$, the interval possibility can be illustrated as shown in Eq. (4):

4 $$ P(a \le b) = \left\{ {\begin{array}{*{20}c} {0,b^{R} \le a^{L} } \\ {\frac{{(b^{R} - a^{L} )}}{{8a^{r} b^{r} }},b^{L} < a^{L} < b^{R} < a^{R} } \\ {\frac{{b^{c} - a^{L} }}{{2a^{r} }},a^{L} < b^{L} < b^{R} \le a^{R} } \\ {1 - \frac{{(a^{R} - b^{L} )}}{{8a^{r} b^{r} }},a^{L} \le b^{L} < a^{R} \le b^{R} } \\ {\frac{{b^{R} - a^{c} }}{{2b^{{\text{r}}} }},b^{L} \le a^{L} < a^{R} \le {\text{b}}^{{}} } \\ 1 \\ \end{array} } \right. $$

In Eq. (4), when $p(a \le b) \ge 0.5$, the number of two-parameter intervals $a \le {\text{b}}$.

Parameter $a$ is set as a three-parameter interval number, which is recorded as $a = [a^{L} ,a^{*} ,a^{R} ]$. Parameter $a^{L}$ is the upper limit of the number of three-parameter intervals, $a^{R}$ is the lower limit of the number of three-parameter intervals, and $a^{*}$ is the center of gravity of the three-parameter interval number. At present, the research of three-parameter interval number focuses on the sorting method of parameter interval number. The existing research is not in-depth, most researchers only consider the parameters, and few consider their uncertainty^16,17^. Here, the three-parameter interval number is transformed into two-parameter interval number, then, the two-parameter interval number possibility method is used for comparison. According to the definition of three-parameter interval number, the two-parameter interval number can be written in the form of midpoint and radius, $a = [a^{L} ,a^{R} ]$ can be converted into the form shown in Eq. (5):

5 $$ a = \left[ {\frac{{a^{L} + a^{R} }}{2} - \frac{{a^{R} - a^{L} }}{2},\frac{{a^{L} + a^{R} }}{2} + \frac{{a^{R} - a^{L} }}{2}} \right] $$

Compared with the two-parameter interval number, the three-parameter interval number has more barycenter information, and adding barycenter information to the center information layer of the interval number can be used to obtain the relationship shown in Eq. (6):

6 $$ a = \left[ {\left( {a^{*} + \frac{{a^{L} { + }a^{R} }}{2}} \right) - \frac{{a^{R} - a^{L} }}{2},\left( {a^{*} + \frac{{a^{L} { + }a^{R} }}{2}} \right) + \frac{{a^{R} - a^{L} }}{2}} \right] $$

In practice, it is necessary to adjust the center of gravity and midpoint information of the two-parameter interval number, and design a weight coefficient as shown in Eq. (7) for the relationship shown in Eq. (5):

7 $$ a = \left[ {\left( {a^{*} + \tau \left( {\frac{{a^{L} { + }a^{R} }}{2}} \right)} \right) - \frac{{a^{R} - a^{L} }}{2},\left( {a^{*} + \tau \left( {\frac{{a^{L} { + }a^{R} }}{2}} \right)} \right) + \frac{{a^{R} - a^{L} }}{2}} \right] $$

### Regret theory

Regret theory can be regarded as a theory of utility function type, which consists of utility function and regret-joy function. The utility function varies with different alternatives^18,19^. If the attribute of *v(x)* is benefit type, its utility function can be written as shown in Eq. (8):

8 $$ v(x) = 1 - \alpha x,0 < \alpha < 1 $$

In Eq. (8), $\alpha$ refers to the risk aversion coefficient for decision makers, concurrently, the utility value of attribute $x$ increases with the increase of risk aversion coefficient of decision makers.

Supposing there are two alternatives.$$A_{1}$$, $$T_{l} = \left[ {\begin{array}{*{20}c} {\varsigma_{11}^{l} } & {\varsigma_{12}^{l} } & \cdots & {\varsigma_{1n}^{l} } \\ {\varsigma_{21}^{l} } & {\varsigma_{22}^{l} } & \cdots & {\varsigma_{2n}^{l} } \\ \vdots & \vdots & \ddots & \vdots \\ {\varsigma_{m1}^{l} } & {\varsigma_{m2}^{l} } & \cdots & {\varsigma_{mn}^{l} } \\ \end{array} } \right]$$$$A_{2}$$, the decision information are referred as:$$x_{1}$$ and $$x_{2}$$. If the decision maker chooses the scheme $$A_{1}$$, instead of $$A_{2}$$, the regret-joy function can be denoted as shown in Eq. (9).

9 $$ R(x_{1} ,x_{2} ) = 1 - exp( - \delta (v(x_{1} ) - v(x_{2} ))) $$

In Eq. (9), $v(x_{1} )$ means the function of the utility of the alternative scheme $A_{1}$, $v(x_{2} )$ stands for the function of the utility of the alternative scheme $A_{2}$, and $\delta$ refers to voidance coefficient for regret. With scheme $A_{2}$ as the reference object, the perception utility function of decision-makers on the alternatives scheme $A_{1}$ can be established as shown in Eq. (10):

10 $$ v(x_{1} ,x_{2} ) = v(x_{1} ) + R(x_{1} ,x_{2} ) $$

### Solution of multi-attribute decision making problem with three parameter interval numbers

Within the research scope, primarily it is supposed that $C = \{ C_{1} ,C_{2} , \ldots C_{n} \}$ is the decision matrix of state $l$, and the probability of occurrence $l$ is $\overline{{P_{l} }}$.In the decision information of different states, $A = \{ A_{1} ,A_{2} , \ldots A_{{\text{m}}} \}$ stands for a collection of schemes, and $C = \{ C_{1} ,C_{2} , \ldots C_{n} \}$ refers to a collection of attributes. The decision matrix of state scheme set A under attribute C can be deduced as shown in Eq. (11).

11 $$ D_{l} = \left[ {\begin{array}{*{20}c} {\xi_{11}^{l} } & {\xi_{12}^{l} } & {...} & {\xi_{1n}^{l} } \\ {\xi_{21}^{l} } & {\xi_{22}^{l} } & {...} & {\xi_{2n}^{l} } \\ \vdots & \vdots & \ddots & \vdots \\ {\xi_{m1}^{l} } & {\xi_{m2}^{l} } & \cdots & {\xi_{mn}^{l} } \\ \end{array} } \right] $$

In Eq. (11), $\xi_{ij}^{l} = [a_{ij}^{L} ,a_{ij}^{*} ,a_{ij}^{R} ](l = 1,2, \ldots ,T;\;i = 1,2 \ldots ,m;\;j = 1,2, \ldots ,n)$ refers to the three-parameter (interval number) decision information of scheme $A_{{\text{i}}}$ in the attributes $C_{j}$ and state *l*.

In terms of the state $l$, the scheme, the decision information of scheme $A_{i}$ under the attributes $C_{j}$ is supposed to be $\xi_{ij}^{l} = [a_{{{\text{ij}}}}^{L} a_{{{\text{ij}}}}^{*} ,a_{{{\text{ij}}}}^{R} ]$. The two-parameter (interval number) information decision matrix $T_{l}$ expressed in Eq. (12) can be obtained by transforming the three-parameter (interval number) into two-parameter interval number.

12 $$ T_{l} = \left[ {\begin{array}{*{20}c} {\varsigma_{11}^{l} } & {\varsigma_{12}^{l} } & \cdots & {\varsigma_{1n}^{l} } \\ {\varsigma_{21}^{l} } & {\varsigma_{22}^{l} } & \cdots & {\varsigma_{2n}^{l} } \\ \vdots & \vdots & \ddots & \vdots \\ {\varsigma_{m1}^{1} } & {\varsigma_{m2}^{1} } & \cdots & {\varsigma_{mn}^{1} } \\ \end{array} } \right] $$

In Eq. (12), $\varsigma_{ij}^{l} = [b_{ij}^{L} ,b_{ij}^{R} ]$ denotes the decision information of the scheme $A_{i}$ under the attribute $C_{j}$ in two-parameter (interval number).

According to different attributes of decision information $\varsigma_{ij}^{l} = [b_{{{\text{i}}j}}^{L} ,b_{ij}^{R} ]$, the two-parameter (interval number) decision matrix is normalized. The relationship presented in Eq. (13) can be found for the benefit attribute.

13 $$ \left\{ {\begin{array}{*{20}c} {r_{{{\text{ij}}}}^{L} { = }\frac{{b_{{{\text{ij}}}}^{L} - \mathop {\min }\limits_{1 \le i \le m} \{ {\text{b}}_{ij}^{L} \} }}{{\max_{1 \le i \le m} \{ {\text{b}}_{ij}^{R} \} - \min_{1 \le i \le m} \{ {\text{b}}_{ij}^{L} \} }}} \\ {r_{{{\text{ij}}}}^{R} { = }\frac{{b_{{{\text{ij}}}}^{R} - \min_{1 \le i \le m} \{ {\text{b}}_{ij}^{L} \} }}{{\max_{1 \le i \le m} \{ {\text{b}}_{ij}^{R} \} - \min_{1 \le i \le m} \{ {\text{b}}_{ij}^{L} \} }}} \\ \end{array} } \right. $$

The normalized interval decision matrix $R_{l}$ is obtained as illustrated in Eq. (14).

14 $$ R_{l} = \left[ {\begin{array}{*{20}c} {\varsigma_{11}^{l} } & {\varsigma_{12}^{l} } & \cdots & {\varsigma_{1n}^{l} } \\ {\varsigma_{21}^{l} } & {\varsigma_{22}^{l} } & \cdots & {\varsigma_{2n}^{l} } \\ \vdots & \vdots & \ddots & \vdots \\ {\varsigma_{m1}^{1} } & {\varsigma_{m2}^{1} } & \cdots & {\varsigma_{mn}^{1} } \\ \end{array} } \right] $$

Among Eq. (14), $\varsigma_{ij}^{l} = [{\text{r}}_{ij}^{L} ,{\text{r}}_{ij}^{R} ]$ refers to the normalized decision information of the scheme $A_{i}$ under the attribute $C_{j}$.

Equation (15) displays the positive ideal scheme matrix.

15 $$ A_{l}^{ + } { = [}\varsigma_{1}^{l + } {,}\varsigma_{2}^{l + } {,} \ldots \varsigma_{n}^{l + } {]} $$

Equation (16) signifies the utility matrix of the attribute.

16 $$ v_{l} = \left[ {\begin{array}{*{20}c} {\chi_{11}^{l} } & {\chi_{12}^{l} } & \cdots & {\chi_{1n}^{l} } \\ {\chi_{21}^{l} } & {\chi_{22}^{l} } & \cdots & {\chi_{2n}^{l} } \\ \vdots & \vdots & \vdots & \vdots \\ {\chi_{m1}^{l} } & {\chi_{m2}^{l} } & \cdots & {\chi_{mn}^{l} } \\ \end{array} } \right] $$

In Eq. (16), $\chi_{ij}^{l} = [{\text{c}}_{ij}^{L} ,{\text{c}}_{ij}^{R} ]$ refers to the wo-parameter (interval number) utility value of scheme $A_{i}$ under the attribute $C_{j}$.

According to the analysis of the research results of other researchers, results show that the idea of pairwise comparison of alternatives is more consistent with the thinking of decision makers. However, there are also problems of high complexity. Therefore, optimization is made. Equation (17)^20,21^ signifies the situation in a regret-joy function where decision makers choose scheme $A_{i}$ other than ideal scheme $A^{ + }$ under the attribute $C_{j}$ of state $l$.

17 $$ R(x_{ij}^{l} ,x_{ij}^{l + } ) = 1 - \exp ( - \delta (v(x_{ij}^{l} ) - v(x_{ij}^{l + } ))) $$

In Eq. (17), $x_{ij}^{l}$ refers to the decision information of scheme $A_{i}$ under the attribute $C_{j}$ in the state $l$, $x_{ij}^{{l{ + }}}$ denotes the decision information of positive ideal scheme $A^{ + }$ under the attribute $C_{j}$ in the state $l$, $v(x_{ij}^{l} )$ signifies the utility value of scheme $A_{i}$ under attribute $C_{j}$ for the decision makers and $v(x_{ij}^{l + } )$ means the utility value of positive ideal scheme $A^{ + }$ under attribute $C_{j}$ for the decision makers.

In the same principle, perceptual utility function can be obtained of scheme $A^{ + }$ under attribute $C_{j}$ in the state $l$ as shown in Eq. (18). Equation (19) presents the perceptual utility matrix, and Eq. (20) signifies the comprehensive perceptual utility matrix.

18 $$ u(x_{ij}^{l} ) = v(x_{ij}^{l} ) + R(x_{ij}^{l} ,x_{j}^{l + } ) $$

19 $$ U_{l} { = }\left[ {\begin{array}{*{20}c} {u_{11}^{{\text{l}}} } & {u_{12}^{{\text{l}}} } & \cdots & {u_{{1{\text{n}}}}^{{\text{l}}} } \\ {u_{21}^{{\text{l}}} } & {u_{22}^{{\text{l}}} } & \cdots & {u_{{2{\text{n}}}}^{{\text{l}}} } \\ \vdots & \vdots & \vdots & \vdots \\ {{\text{u}}_{m1}^{{\text{l}}} } & {{\text{u}}_{m2}^{{\text{l}}} } & \cdots & {{\text{u}}_{mn}^{{\text{l}}} } \\ \end{array} } \right] $$

In Eq. (19), $u_{ij}^{l} = [{\text{d}}_{ij}^{L} ,{\text{d}}_{ij}^{R} ]$ denotes utility value of the scheme $A_{i}$ under attribute $C_{j}$ in state $l$.

20 $$ U = \left[ {\begin{array}{*{20}c} {\varphi_{11} } & {\varphi_{12} } & \cdots & {\varphi_{{1{\text{n}}}} } \\ {\varphi_{21} } & {\varphi_{22} } & \cdots & {\varphi_{{{\text{2n}}}} } \\ \vdots & \vdots & \vdots & \vdots \\ {\varphi_{{{\text{m}}1}} } & {\varphi_{{{\text{m2}}}} } & {...} & {\varphi_{{{\text{mn}}}} } \\ \end{array} } \right] $$

In Eq. (20), $\varphi_{ij} = [\varphi_{ij}^{L} ,\varphi_{ij}^{R} ] = \sum\nolimits_{l}^{t} {u_{ij}^{l} } \times \overline{P}_{l} = \sum\nolimits_{l = 1}^{t} {([d_{{{\text{ij}}}}^{L} ,d_{{{\text{ij}}}}^{R} ] \times \overline{P}_{l} )}$ stands for the comprehensive perceptual utility value of the scheme $A_{i}$ in the attribute $C_{j}$, and $\overline{P}_{l}$ refers to the probability of occurrence of state $l$.

The comprehensive utility matrix of alternative scheme $A_{i}$ can be obtained as shown in Eq. (21).

21 $$ U^{^{\prime}} = \left[ {\begin{array}{*{20}c} {\gamma_{1} } \\ {\gamma_{2} } \\ \vdots \\ {\gamma_{{\text{m}}} } \\ \end{array} } \right] $$

Equation (21) $\gamma_{{\text{i}}}$ can also be written as Eq. (22):

22 $$ \gamma_{i} = [\gamma_{i}^{L} ,\gamma_{i}^{R} ] = \sum\limits_{j = 1}^{n} {\varphi_{ij} } \omega_{j} = \sum\limits_{j = 1}^{{\text{n}}} {\left( {\left[ {\varphi_{{{\text{ij}}}}^{L} ,\varphi_{{{\text{ij}}}}^{R} } \right] \times \omega_{j} } \right)} $$

In Eq. (22), $\omega_{j}$ denotes the weight of attribute $C_{j}$.

Ultimately, the ranking vector $\theta = (\theta_{1} ,\theta_{2} , \ldots \theta_{{\text{m}}} )^{T}$ of the probability matrix p is obtained by using the ranking Eq. (22). And the vectors $\theta_{{\text{i}}}$ are sorted to determine the scheme.

### Solution of attribute weight in multi-attribute decision making

According to the corresponding literature, Eq. (23) presents the priority weight of the comprehensive perceived utility value related to the scheme $A_{i}$ in the attribute $C_{j}$.

23 $$ W = \left[ {\begin{array}{*{20}c} {w_{11} } & {w_{12} } & \cdots & {w_{1n} } \\ {w_{21} } & {w_{22} } & \cdots & {w_{2n} } \\ \vdots & \vdots & \vdots & \vdots \\ {w_{m1} } & {w_{m2} } & \cdots & {w_{mn} } \\ \end{array} } \right] $$

In Eq. (23), $w_{ij}$ represents the weight of attribute $C_{j}$ of scheme $A_{i}$.

From $\Gamma = (UW^{T} )^{T} (UW^{T} ) = (WU^{T} )(W^{T} U)$, normalized eigenvectors $\rho = [\rho_{1} \rho_{2} \ldots \rho_{m} ]^{T}$ of $\Gamma$ can be obtained. The discrete projection factor of alternative scheme is set as $\rho = [\rho_{1} \rho_{2} \ldots \rho_{m} ]^{T}$, then, Eq. (24) shows the calculation of the weight of attribute.

24 $$ \omega = W^{T} \rho = \left[ {\begin{array}{*{20}c} {\omega_{11} } & {\omega_{21} } & \cdots & {\omega_{m1} } \\ {\omega_{12} } & {\omega_{22} } & \cdots & {\omega_{m2} } \\ \vdots & \vdots & \vdots & \vdots \\ {\omega_{1n} } & {\omega_{2n} } & \cdots & {\omega_{mn} } \\ \end{array} } \right]\left[ {\begin{array}{*{20}c} {\rho_{1} } \\ {\rho_{2} } \\ \vdots \\ {\rho_{m} } \\ \end{array} } \right] $$

Generally speaking, in the actual decision-making process, the decision-maker can only give a reasonable range of attribute preference according to limited information and personal subjective preference^22–24^.

### Optimization of mixed multi-criteria decision-making with large samples

Since the publication of *Game Theory and Economic Behavior* by economist VonNeumann and mathematician OskarMorgenstem in the 1940s, the expected utility theory has become the main guiding theory in the economics and laid the foundation for multi-criteria decision-making field. The expected utility theory is characterized by supposing people as "completely rational". However, as the economic and social situation becomes more and more complex, the decision-making environment becomes more and more diverse, and the uncertainty is obviously enhanced^25,26^. The decision information obtained by decision makers is not comprehensive, and the "risk decision problem" has attracted more and more people's attention, but it is not consistent with the expected utility theory. In view of this contradictory phenomenon, French economists put forward Alai paradox, which overturns the viewpoint that the utility function is linear in the expected utility theory^27,28^. In the late 1970s, two researchers put forward a new kind of risk decision theory, namely prospect theory, which used prospect instead of utility. The feature of prospect theory is that people's decision-making process is divided into two stages: the first stage is to collect and sort out the evaluation objects, and the second stage is to integrate the data collected in the first stage according to certain methods, so as to make decisions and evaluations. Intermittent scheme $A_{i}$ can be written as Eq. (25)^29,30^.

25 $$ V_{i} = \sum\limits_{j = 1}^{n} {\pi (p)v(\Delta x)} $$

In Eq. (25), $\pi (p)$ refers to the decision weight function, $\Delta x$ is the value function, in addition to the value function. $\Delta x$ include reference points.$\theta_{j}$ And risk factor.$\alpha ,\beta ,\lambda$ the expression of power function is shown in Eq. (26):

26 $$ v(\Delta x) = \left\{ {\begin{array}{*{20}c} {\Delta x^{\alpha } ,\quad \quad \;\;\;x_{ij} \succ \theta_{j} } \\ { - \lambda (\Delta x)^{\beta } ,\quad x_{ij} \prec \theta_{j} } \\ \end{array} } \right. $$

In Eq. (26), if $x_{ij} \succ \theta_{j}$, profits lie in $x_{ij}$ compared to the reference point $\theta_{j}$. On the contrary, if $x_{ij} \prec \theta_{j}$, there are profits lying in $x_{ij}$ compared to the reference point $\theta_{j}$. Coefficient $\alpha ,\beta ,\lambda$ stand for degree of risk preference, risk aversion and risk aversion coefficient, separately. $\Delta x$ refers to the distance of $x_{ij}$ to the reference point $\theta_{j}$. Equation (27) denotes the decision weight function $\pi (p)$.

27 $$ \pi (p) = \frac{{p^{\gamma } }}{{(p^{\gamma } + (1 - p)^{\gamma } )^{{{\raise0.7ex\hbox{$1$} \!\mathord{\left/ {\vphantom {1 \lambda }}\right.\kern-\nulldelimiterspace} \!\lower0.7ex\hbox{$\lambda $}}}} }} $$

Equation (28) expresses the typical linear weighting equation in the multi-criteria decision-making problem discussed here.

28 $$ U{}_{i} = \sum\limits_{j = 1}^{n} {w_{j} v_{ij} } $$

In Eq. (28), $v_{{{\text{ij}}}}$ means prospect value of scheme $A_{i}$ in the attribute $C_{j}$, and $w_{j}$ denotes the criterion of attribute $C_{j}$.

**Figure 3:**
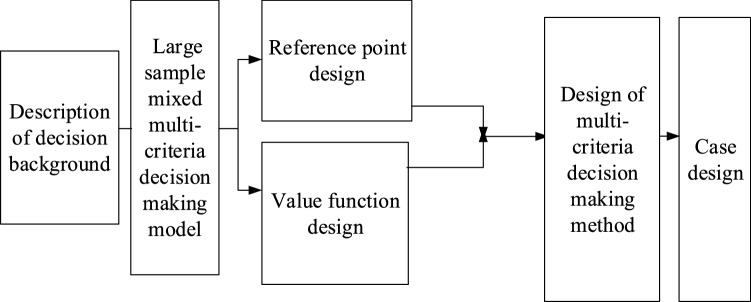
optimization of the prospect theory.

The key point of the prospect theory is the reference point, and the change of the reference point will affect the decision makers' evaluation of the same scheme. Study is made on the mixed multi-criteria decision-making problem with large samples, in other words, there are many kinds of decision-making schemes, and different evaluation criteria have different values^31,32^. According to the actual situation, an objective data class is used to obtain reference points, and a nonparametric distribution fitting algorithm is designed. Figure 3 displays the research idea.

In the research scope, the decision scheme set is set as $A = \{ A_{1} ,A_{2} , \ldots A_{m} \}$, the criterion is written as $C = \{ C_{1} ,C_{2} , \ldots C_{n} \}$, and the weight of criteria $C{}_{j}$ is made as $w{}_{j}$ with $W = (w_{1} ,w_{2} , \ldots w_{n} )^{T}$. Criterion $C{}_{j}$ meets $\sum w_{j} = 1,\;j = 1,2, \ldots ,n$.

Parameter $x_{ij}$ refers to the value of scheme $A_{{\text{i}}}$ in the criterion. In the comparison between $x_{ij}$ and the reference point $\theta_{j}$ of criterion $C{}_{j}$, when $x_{ij}$ performances better than $\theta_{j}$ good, it is considered to have a positive effect; otherwise, it has a negative effect. The distance between $x_{ij}$ and $\theta_{j}$ refers to how much to gain or suffer. The discrete points are plotted as sample distribution curves, and the characteristics of the curves are analyzed, and the curves are fitted to deduce assumptions, and nonparametric tests are carried out to obtain the expected values. Its specific process can be divided into three steps:

1. The sample data with capacity “a” of criterion $C{}_{j}$ is plotted as a graph line to judge the theoretical distribution types of $F_{a} (x)$. And sample data is utilized to calculate parameters and construct assumptions as Eqs. (29) and (30).
  29 $$ H_{0} :F_{a} (x) = F(x) $$
  30 $$ H_{1} :F_{a} (x) \ne F(x) $$

In Eq. (30), $d_{i}$ refers to the absolute difference between the cumulative frequency and the cumulative distribution frequency of samples, $x_{(i)}$ means the statistics of orders of samples, among which the largest absolute difference $D{}_{a}$ can be written as Eq. (31).

31 $$ \begin{aligned} D{}_{a} = & \max \{ \left| {F_{a} (x) - F(x)} \right|\} \\ = & \max \{ d_{1} ,d_{2} , \ldots d_{a} \} \\ \end{aligned} $$

2. When the original hypothesis holds, the value of $D{}_{a}$ is relatively mall. When the sample size $a < 100$, the critical value $D_{(m,a)}$ can be directly looked up in the table. When the sample size $a \ge 100$, parameter $D_{(m,a)}$ can be expressed as Eq. (32).
  32 $$ D_{(m,a)} = \frac{{\lambda_{1 - \alpha } }}{\sqrt m } $$
3. Values of $D{}_{a}$ and $D_{(m,a)}$ are compared, when $D{}_{a}$ is smaller than $D_{(m,a)}$, the original hypothesis holds, otherwise, other types of distribution will be selected. In the latter condition, the operations on the hypothesis constructed above will be repeated until a reasonable distribution type occurs.

The design of the value function is based on the idea of distance and possibility. Equation (33) can fully illustrate the judgment rule of parameter $x_{ij}$ and parameter $\theta_{j}$.

33 $$ \Delta x_{ij} = \left| {x_{{_{ij} }} - \theta_{j} } \right| $$

When $x_{{_{ij} }} - \theta_{j} \ge 0$, then $x_{{_{ij} }} \succ \theta_{j}$, and vice versa.

## Example analysis

### Case analysis of H1NI epidemic

Taking the epidemic response decision-making problem as an example, the feasibility of the decision-making method is verified. A school found that there is a risk of the spread of the H1NI epidemic, and there are three response plans $$A_{1} ,A_{2} ,A_{3}$$. Through communication with experts, it is found that there are three symbolic result scenarios $$S_{1} ,S_{2} ,S_{3}$$ for the spread of the H1NI epidemic. The attribute set is the number of infected people $$C_{1}$$, economic loss $$C_{2}$$, social impact $$C_{3}$$, and each attribute weight vector $$\omega = (0.6,0.2,0.2)$$. The decision maker's expectation vector $$E = ([20,25],[25,30],s_{3} )[25,30]$$$$,s_{3} )$$ for each attribute value under different scenarios, the attribute values and their priors of the result scenarios under different scenarios are shown in Table 1.

**Table 1:** The attribute values and priors of the result scenarios under different schemes.

Scenario	$C_{1}$	$C_{2}$	$C_{3}$	$P(S_{j} |A_{i} )$	$S_{1}$	$S_{2}$	$S_{3}$
$S_{1}$	[5, 10]	[5, 10]	$S_{4}$	$A_{1}$	0.5	0.3	0.15
$S_{2}$	[11, 20]	[20, 30]	$S_{2}$	$A_{2}$	0.6	0.25	0.20
$S_{3}$	[21,40]	[40,60]	$S_{0}$	$A_{3}$	0.70	0.20	0.10

According to Table 1, this type of decision information structure has the same results but different probabilities, and its normalized results are shown in Table 2.

**Table 2:** Values of the matrixes of normalized schemes.

Scenario	$S_{1}$			$S_{2}$			$S_{3}$		
	$C_{1}$	$C_{2}$	$C_{3}$	$C_{1}$	$C_{2}$	$C_{3}$	$C_{1}$	$C_{2}$	$C_{3}$
$A_{1}$	[0.44,0.72]	0.73	[0,0.17,0.33]	[0.28,0.44]	0.42	[0.17,0.33,0.5]	[0.06,0.21]	0.17	[0.67,0.83,1]
$A_{2}$	[0.71,1]	0.89	[0.17,0.33,0.56]	[0.33,0.61]	0.52	[0.33,0.5,0.67]	[0.17,0.39]	0.38	[0.67,0.83,1]
$A_{3}$	[0.5,0.83]	0.83	[0.0.17,0.33]	[0.17,0.39]	0.31	[0.17,0.33,0.5]	[0,0.17]	0	[0.33,0.5,1]

Conclusion can be drawn from calculations of the above figures that, the overall utility uncertainty degree of psychological perception is calculated.$H_{1}^{^{\prime}} = 0.101$, $H_{2}^{^{\prime}} = 0.081$, $H_{3}^{^{\prime}} = 0.091$, and the order of the scheme can be determined as:$H_{2}$, $H_{3}$, $H_{1}$.

### Analysis of an example in COVID-19

**Figure 4:**
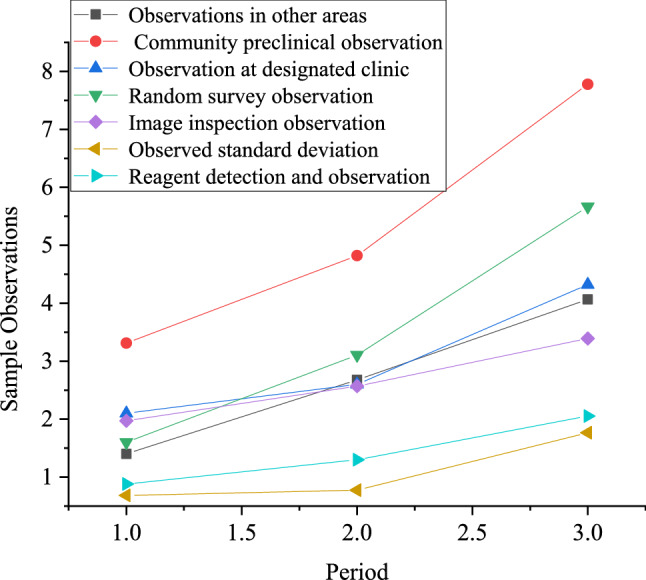
Six observation sample values of infection rate in COVID-19.

The data of the infection rate of COVID-19 epidemic on January 24th is taken as prior information to stimulate the multi-stage dynamic decision-making for 4 days from January 25th to 28th after Wuhan was closed. There are about 9 million people in Wuhan, and about 5 million people have moved out. According to the calculation, the infection rate of COVID-19 in Wuhan on January 24th is 0.63%, and that in other parts of China is 1.4040%. The two infection rates are quite different. The mean and standard deviation of these two data are taken as the prior mean and prior standard deviation of actual infection rate $\theta_{1}$ in Wuhan on January 25th, where prior distribution of infection rate $\theta_{1}$ is supposed to meet N (1.0198,0.5434). Figure 4 illustrates six kinds of observation sample values of COVID-19 infection rate of every day in Wuhan.

**Figure 5:**
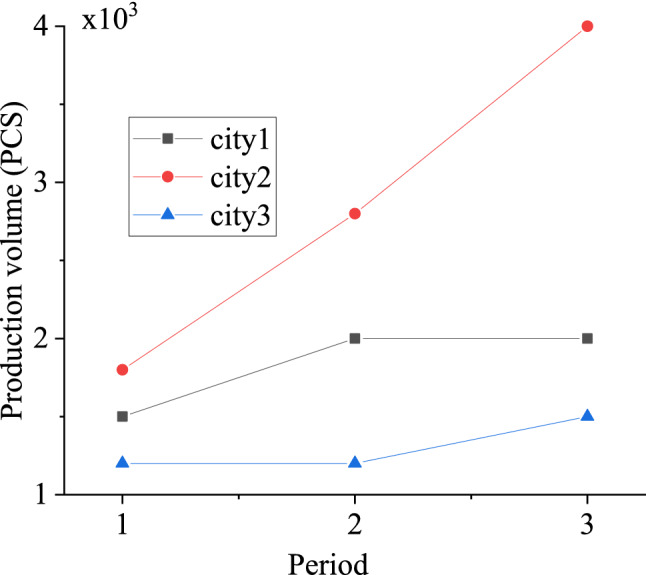
Mask supply of 3 manufacturers in 3 cities.

Assuming that the demand for masks in one case is 10, there are three types of supply sources: central reserve, donation and production enterprises. Assuming that the total central reserve is 10,000, and the number of donations is uncertain. Figure 5 denotes the daily supply of three production enterprises in three cities.

MATLAB is used to program the algorithm proposed here, and the optimal decision time is 0 every day, namely, the emergency material allocation decision is made as early as possible according to the posterior mean of the infection rate of the previous day before the observation. This is because although the uncertainty of infection rate is relatively large, the uncertainty of observation is even greater. The marginal delay loss of decision is greater than the marginal error loss. The daily optimal emergency material allocation scheme is divided into three cycles. In cycle 1, the optimal total allocation amount is 9178, of which the donation amount is 788, the central reserve allocation amount is 8380, and the production allocation amount of the production enterprise is 0. In period 2, the optimal total allocation amount is 14,372.Among them, the donation amount is 4853, the central reserve allocation amount is 1610, and the production allocation amount of production enterprises is 7909. In the period of cycle 3, the optimal total allocation amount is 19,011, of which the donation amount is 2453, the central reserve allocation amount is 0, and the production allocation amount of production enterprises is 12,500, which cannot meet the actual demand of Wuhan area. From the above data, it can be seen that the central reserve cannot be continuously supplied. As donation is random, production becomes the basis of ensuring the continuous and stable supply of emergency materials. When shortages occur in cycle 3, it is required of urgent international procurement or further expansion of production capacity.

**Figure 6:**
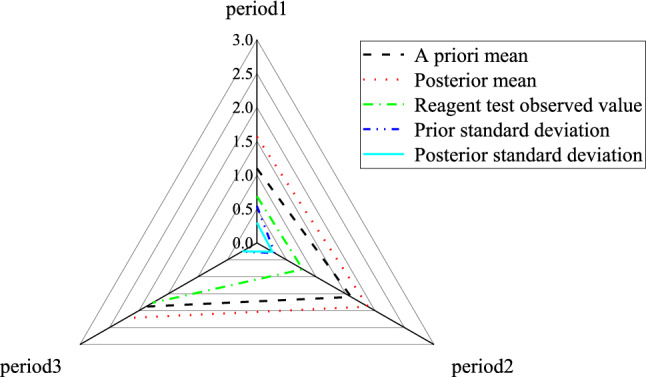
Statistics of infection rate.

**Figure 7:**
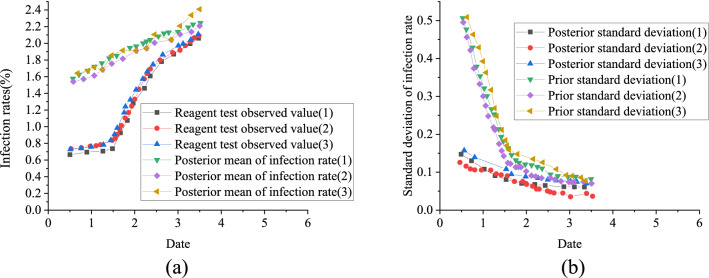
Statistical comparison of infection rate (**a**) the posterior mean of infection rate and the observation value of reagent detection; (**b**) the comparison of prior standard deviation and posterior standard deviation.

Figure 6 reveals the statistics of daily infection rate. The post validation value of infection rate is compared with the observation value of reagent detection, of which Fig. 7 displays the results.

Figure 7 signifies that the posterior mean curve of infection rate is smoother than the observation value of reagent detection, which can show that it is not accurate to detect the infection rate only by reagent. However, when the infection rate in other areas, community outpatient service, fixed-point outpatient service and image inspection are considered and updated accordingly, the time to obtain the relative real infection rate information will be advanced. After the information is updated, the standard deviation of infection rate will gradually decrease, and the uncertainty of epidemic information will be reduced.

**Figure 8:**
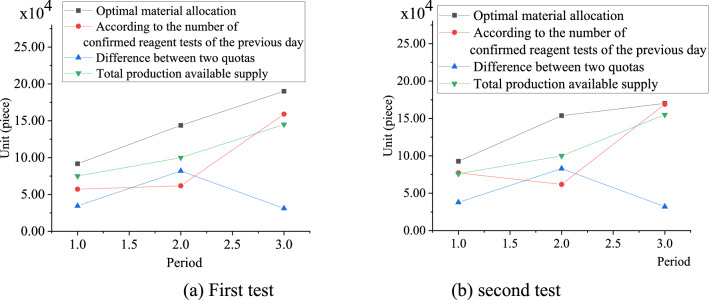
Allocation and supply of emergency materials.

Figure 8 presents the situations of material allocation and production supply in COVID-19 emergency.

Figure 8 displays the allocation of emergency materials and production supply. The optimal material allocation amount obtained by this model is larger than that calculated by the number of confirmed reagent detection cases the previous day, which can more effectively meet the actual demand for emergency supplies in Wuhan and reduce the infection of medical staff.

**Figure 9:**
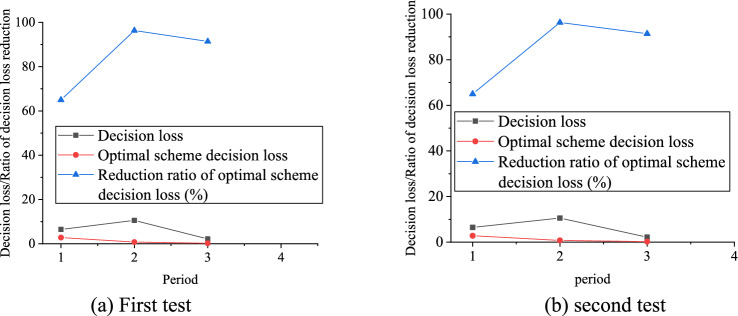
Decision loss of emergency material allocation scheme.

Figure 9 displays the decision loss of emergency material allocation scheme.

The emergency material configuration scheme is tested twice, and the analysis is carried out with Fig. 9a as an example. As Fig. 9a shows, when the epidemic situation is in the first cycle, the optimal scheme decision-making loss is 2.69, and the reduction proportion of the optimal scheme decision-making loss is 63.96%; when the epidemic situation is in the second cycle, the decision-making loss of the optimal scheme is 0.65, and the reduction ratio of the optimal scheme decision-making loss is 94.44%. When the epidemic situation is in the third cycle, the decision-making loss of the optimal scheme is 0.22, and the reduction ratio of the decision-making loss of the optimal scheme is 89.39%. From the decision-making loss of the emergency material allocation scheme, results imply that the decision-making loss can be greatly reduced by the optimal material allocation scheme compared with the allocation according to the number of reagent detection confirmed the previous day.

**Figure 10:**
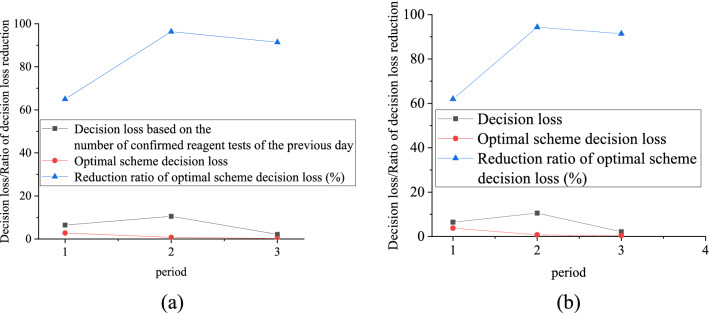
Comparison of decision-making losses in emergency material allocation plans. (**a**) The proposed algorithm; (**b**) The algorithm proposed in^33^).

The decision loss of the designed emergency material allocation scheme and the decision loss of the emergency material allocation scheme designed by Siddharth et al.^33^ are shown in Fig. 10.

Figure 10a indicates that when the epidemic situation is in the first cycle, the decision-making loss of the proposed scheme is 2.69, and the reduction ratio of the decision-making loss is 63.96%. When the epidemic situation is in the second cycle, the decision-making loss of the proposed scheme is 0.65, and the reduction ratio of the decision-making loss is 94.44%. When the epidemic situation is in the third cycle, the decision-making loss of the proposed scheme is 0.22, and the reduction ratio of the decision-making loss is 89.39%. Figure 10b denotes that when the epidemic situation is in the first cycle, the decision loss of the proposed scheme in^33^ is 3.69, and the reduction ratio of the decision-making loss is 68.96%. When the epidemic situation is in the second cycle, the decision loss of the proposed scheme by^33^ is 0.85, and the reduction ratio of the optimal scheme decision-making loss is 92.44%. When the epidemic situation is in the third cycle, the decision loss of the proposed scheme in^33^ is 0.42, and the reduction ratio of the decision-making loss of the optimal scheme is 89.19%. In contrast, the new designed scheme can reduce the decision-making loss.

## Conclusions

Firstly, starting from the processing method of fuzzy numbers, it focuses on the typical fuzzy multi-criteria decision-making problem. Through the combination of prospect theory and multi-criteria decision-making method, the shortcomings of each other can be made up and the advantages of each other can be fully utilized. Secondly, the risks of different stages of the development and evolution of major public health emergencies are analyzed. According to the perceived utility value of different levels of risk, the main decision-making members are perceived as utility, and a utility matrix is established at the same time. The weight of decision-making members is calculated and revised according to the results of utility clustering, and the decision-making scheme is determined according to the clustering results. The risk decision-making model is constructed by combining the utility risk entropy algorithm and prospect theory. The model is used to conduct case analysis, calculate the conflict risk entropy of different types of health safety accidents. Combined with utility perception, the emergency plan is prioritized, thereby improving the processing efficiency of major health emergencies and reducing the risk of accidents. Finally, the proposed scheme is tested, and the test results demonstrate that when the epidemic situation is in the first cycle, the decision-making loss of the proposed scheme is 2.69, and the reduction ratio of the decision-making loss is 63.96%. When the epidemic situation is in the second cycle, the decision-making loss of the proposed is 0.65, and the reduction ratio of the decision-making loss is 94.44%. When the epidemic situation is in the third cycle, the decision-making loss of the proposed is 0.22, and the reduction ratio of the decision-making loss is 89.39%. Compared with the results of other researchers, the proposed algorithm has less loss and faster response speed.

However, the proposed scheme still has some deficiencies. The proposed method has many limitations and is mainly aimed at the situation of large samples, and there is no further analysis of the influence of the number of samples on the decision-making results. These deficiencies will continue to be studied in the follow-up work.
